# CRISPR/Cas9-mediated genome-edited mice reveal 10 testis-enriched genes are dispensable for male fecundity

**DOI:** 10.1093/biolre/ioaa084

**Published:** 2020-05-26

**Authors:** Soojin Park, Keisuke Shimada, Yoshitaka Fujihara, Zoulan Xu, Kentaro Shimada, Tamara Larasati, Putri Pratiwi, Ryan M Matzuk, Darius J Devlin, Zhifeng Yu, Thomas X Garcia, Martin M Matzuk, Masahito Ikawa

**Affiliations:** 1 Graduate School of Medicine, Osaka University, Osaka, Japan; 2 Department of Experimental Genome Research, Research Institute for Microbial Diseases, Osaka University, Osaka, Japan; 3 Department of Bioscience and Genetics, National Cerebral and Cardiovascular Center, Osaka, Japan; 4 Graduate School of Pharmaceutical Sciences, Osaka University, Osaka, Japan; 5 Research Institute for Microbial Diseases, Osaka University, Osaka, Japan; 6 Center for Drug Discovery, Baylor College of Medicine, Houston, TX, USA; 7 Department of Pathology & Immunology, Baylor College of Medicine, Houston, TX, USA; 8 Interdepartmental Program in Translational Biology and Molecular Medicine, Baylor College of Medicine, Houston, TX, USA; 9 Department of Biology and Biotechnology, University of Houston-Clear Lake, Houston, TX, USA; 10 The Institute of Medical Science, The University of Tokyo, Tokyo, Japan

**Keywords:** male infertility, contraception, CRISPR/Cas9, knockout model

## Abstract

As the world population continues to increase to unsustainable levels, the importance of birth control and the development of new contraceptives are emerging. To date, male contraceptive options have been lagging behind those available to women, and those few options available are not satisfactory to everyone. To solve this problem, we have been searching for new candidate target proteins for non-hormonal contraceptives. Testis-specific proteins are appealing targets for male contraceptives because they are more likely to be involved in male reproduction and their targeting by small molecules is predicted to have no on-target harmful effects on other organs. Using in silico analysis, we identified *Erich2*, *Glt6d1*, *Prss58*, *Slfnl1*, *Sppl2c*, *Stpg3*, *Tex33*, and *Tex36* as testis-abundant genes in both mouse and human. The genes, *4930402F06Rik* and *4930568D16Rik*, are testis-abundant paralogs of *Glt6d1* that we also discovered in mice but not in human, and were also included in our studies to eliminate the potential compensation. We generated knockout (KO) mouse lines of all listed genes using the CRISPR/Cas9 system. Analysis of all of the individual KO mouse lines as well as *Glt6d1/4930402F06Rik*/*4930568D16Rik* TKO mouse lines revealed that they are male fertile with no observable defects in reproductive organs, suggesting that these 10 genes are not required for male fertility nor play redundant roles in the case of the 3 *Glt6D1* paralogs. Further studies are needed to uncover protein function(s), but in vivo functional screening using the CRISPR/Cas9 system is a fast and accurate way to find genes essential for male fertility, which may apply to studies of genes expressed elsewhere. In this study, although we could not find any potential protein targets for non-hormonal male contraceptives, our findings help to streamline efforts to find and focus on only the essential genes.

## Teaser

Ten testis-enriched genes are dispensable for male fertility, as determined by phenotypic analyses of knockout mice.

## Introduction

With the global population exceeding 7.7 billion people, contraceptive methods are critical for both personal and societal needs. Accordingly, effective hormonal-based female contraceptives were developed to prevent unintended pregnancy; however, some women are unable to use these contraceptives due to side effects or other health conditions [1]. Meanwhile, a growing number of men are willing to take active responsibility in family planning [2]. Heretofore, effective male contraceptives include condoms or vasectomy, but these choices are viewed as undesirable and inadequate for many and not reversible in the case of vasectomy [3]. The development of novel non-hormonal male contraceptive drugs is anticipated to address some of these issues, but of the few potential non-hormonal male contraceptive drugs in the discovery phase, several have drawbacks for men and women [4].

To find new targets for non-hormonal male contraceptive drugs, a greater emphasis needs to be placed on identifying proteins involved in reproduction. Conceptually, testis-specific proteins offer attractive targets for male contraceptives, due to their potential function in male reproduction and because their disruptions are predicted to have few adverse effects in other organs. We utilized the mouse in our search for potential protein targets because the protein-coding regions of the mouse and human genomes are 85% identical on average [5] and because the mouse provides us an in vivo system to study the effects of the absence of the target protein. We should not forget that no culture systems have been developed that can produce fully functional spermatozoa in vitro. Proteins that are essential for male fertility in our system could then serve as novel targets for non-hormonal male contractive drugs. In addition, our findings may lead to a new treatment for infertile men.

Our laboratories have been using the CRISPR/Cas9 system to generate mice that lack testis-specific genes, and our studies have revealed that several genes are indispensable for male fecundity [6–11]. On the other hand, we reported about 100 testis-enriched genes that are not essential for male fertility [12–15]. In the same context, we generated ten knockout (KO) mouse lines in the present study. We selected eight genes: *Erich2*, *Glt6d1*, *Prss58*, *Slfnl1*, *Sppl2c*, *Stpg3*, *Tex33,* and *Tex36*, which have human orthologs and show testis-enriched expression according to the Mouse ENCODE Project [16]. Among them, *Glt6d1* has two paralogs named *4930402F06Rik* and *4930568D16Rik* in mice, but these paralogs are not found in humans. Because these paralog proteins may compensate for the function of GLT6D1 in mice, we generated double KO (DKO) and triple KO (TKO) mice in the present study. Using these CRISPR/Cas9 genome-edited KO mouse lines, we checked their effects on male reproduction to find candidate target proteins for non-hormonal male contraception.

## Materials and methods

### Animals

All animal experiments performed in this study were approved by the Institutional Animal Care and Use Committees of Osaka University (Osaka, Japan) in compliance with the guidelines and regulations for animal experiments (approval code: H30–01-0; approval date: 4 July 2018). All B6D2F1 and ICR mice were purchased from CLEA Japan, Inc. (Tokyo, Japan) or Japan SLC, Inc. (Shizuoka, Japan). The authors confirm the guidelines as stipulated by the Care and Use of Experimental Animals section of BOR’s instructions to authors were followed.

### Digital PCR

Digital PCR was conducted as previously described [14, 15]. Briefly, sequences for different tissues were downloaded from sequence read archives (SRA, https://www.ncbi.nlm.nih.gov/sra), trimmed using TrimGalore (https://www.bioinformatics.babraham.ac.uk/projects/trim_galore/), and aligned to the human genome (GRCh38) or mouse genome (GRCm38) using HISAT2 [17]. Feature Counts was used to quantify gene expression in each tissue, and RUVr [18] was used to batch correct tissues by removing unwanted variation. EdgeR was used to determine differential gene expression for each non-reproductive tissue against each reproductive tissue [19].

### Phylogenetic tree

The gene tree for each gene was made with GENETYX parallel editor (Genetyx corporation, Tokyo, Japan), ClustalW-2.1 [20], and GENETYX-Tree with the amino acid sequence of human, mouse, chimpanzee, cattle, and dog if orthologs were present. For constructing the phylogenetic tree of GLT6D1, mouse paralog proteins 4930568D16Rik and 4930402F06Rik were included. The amino acid sequences used in this study are listed in Supplementary Table S1.

### Comparison of amino acid sequences

We compared the identity of mouse and human amino acid sequences. Each amino acid sequence (listed in Supplementary Table S1) was submitted to ClustalW to obtain the alignment file. The file was then submitted to JalView [21] to compare the identity.

### Generation of knockout mice using the CRISPR/Cas9 system

All KO mouse lines in this study were produced with the CRISPR/Cas9 genome editing system, and CRISPRdirect software [22] was used to avoid the off-target possibilities. Both *Glt6d1* KO and *4930568D16Rik* KO mouse lines were generated by microinjection of pX330 plasmids (#42230, Addgene, Cambridge, MA, USA) expressing a chimeric guide RNA together with human codon-optimized Cas9 (hCas9) into the pronuclei of zygotes as described previously [23]. The guide RNA sequences are listed in Supplementary Table S2. After injection of plasmid DNA, eggs were cultured in KSOM medium overnight and transferred into the oviducts of pseudopregnant ICR females at 0.5 days after mating with vasectomized males. Pups were obtained by natural or Caesarean section, and subsequent sibling crosses were performed to obtain homozygous KO mice. For making *Glt6d1/4930568D16Rik* DKO mice, *Glt6d1* single KO mice were mated with *4930568D16Rik* single KO mice.

To generate KO mice targeting *Erich2, Slfnl1, Sppl2c, Stpg3, Tex33*, and *Tex36*, we performed electroporation using zygotes as described previously [24]. Two crRNAs listed in Supplementary Table S3 and tracrRNA (Merck, Darmstadt, Germany) were mixed with Cas9 protein (Thermo Fisher Scientific, Waltham, MA, USA) and Opti-MEM (Thermo Fisher Scientific). This solution was incubated in 37°C to make the guide RNA/Cas9 ribonucleoprotein (RNP) complex and the obtained complex was electroporated into fertilized oocytes using NEPA21 Super Electroporator (Nepagene, Chiba, Japan). The treated embryos developed to a two-cell stage were transplanted into the oviducts of pseudo-pregnant female ICR recipients at 0.5 days after mating with vasectomized males. Pups were obtained by natural or Caesarean section, and subsequent sibling crosses were performed to obtain homozygous KO mice. For generating *Glt6d1*/*4930568D16Rik*/*4930402F06Rik* TKO mice, electroporation described above were performed using fertilized oocytes obtained from in vitro fertilization (IVF) using spermatozoa and oocytes obtained from DKO mice.

*Prss58* KO mice were produced using embryonic stem (ES) cells, using methods as previously described [25]. We designed guide RNAs as listed in Supplementary Table S4 and inserted the sequence into the pX459 V2.0 plasmid (#62988, Addgene). The EGR-G101 ES cells [26] were co-transfected with two guide RNA-inserted vectors using Lipofectamine LTX & PLUS reagent (Thermo Fisher Scientific). After selecting with puromycin and genotyping, the mutant ES clones with normal karyotypes were aggregated with 8-cell to morula stage ICR embryos. They were cultured until blastocyst stage and transplanted into the uterus of pseudo-pregnant female ICR recipients at 2.5 days after mating with vasectomized males. Generated chimeric male mice were mated with B6D2F1 females for germline transmission, and subsequent sibling crosses were performed to obtain homozygous KO mice. The primers and PCR conditions for genotyping are listed in Supplementary Table S5.

### Morphological and histological analysis of testis and epididymis

After euthanasia, testes and epididymides were dissected. After measuring the testicular weight, testes, caput and cauda epididymides were fixed in Bouin’s fluid (Polysciences, Inc., Warrington, PA, USA), and embedded in paraffin, sectioned rehydrated and treated with 1% periodic acid for 10 min, followed by treatment with Schiff’s reagent (FUJIFILM Wako, Osaka, Japan) for 20 min. The sections were stained with Mayer’s hematoxylin solution (FUJIFILM Wako) prior to imaging and observed using an Olympus BX53 differential interference contrast microscope equipped with an Olympus DP74 color camera (Olympus, Tokyo, Japan).

### Morphological analysis of spermatozoa and sperm motility

Three male mice of each KO line and age-matched controls (heterozygous mutant) were used in this study. Cauda epididymal spermatozoa were suspended in the TYH medium [27]. A sperm suspension was placed on MA-coated glass slide (Matsunami Glass, Osaka, Japan) and observed using an Olympus BX53 microscope. Sperm motility of cauda epididymal spermatozoa suspended in TYH medium was measured using the CEROS II sperm analysis system (software version 1.5; Hamilton Thorne Biosciences, Beverly, MA, USA) after 10 min and 2 h of incubation.

### Fertility analysis of KO lines

Sexually mature B6D2F1 wild-type (WT) or KO male mice were housed individually with three 6-week-old female B6D2F1 mice for at least 8 weeks. Male mice were removed after the mating period listed in Table 1, and females were kept for another 3 weeks to count the final litters. The numbers of pups and copulation plugs were counted every weekday morning.

**Table 1:** Outcomes of fertility tests for 11 KO mouse lines. Statistical analyses of average litter size between wild-type and each KO mouse line were performed using the Student’s two-tailed t-test for unpaired observations, but no significant differences were detected.

Gene	KO strategy	Genotype	Average litter size ± SD	No. of males	No. of delivery	No. of pups	No. of plugs	Mating period
Wild type	−	−	8.9 ± 0.6	3	24	213	24	8 wks
*4930568D16Rik*	Plasmid inj	−10/−10	7.3 ± 1.7	3	12	88	ND	13 wks
*Erich2*	EP	−31536/−31536	9.8 ± 1.3	3	21	206	21	8 wks
*Glt6d1*	Plasmid inj	−8/−8	9.3 ± 2.0	3	21	195	ND	13 wks
*Glt6d1/4930568D16Rik* (DKO)	Mating	−8/−8 (*Glt6d1*), −10/−10 (*D16Rik*)	9.6 ± 2.1	2	7	67	ND	9 wks
*Glt6d1/4930568D16Rik/4930402F06Rik* (TKO)	EP	−8/−8 (*Glt6d1*), −10/−10 (*D16Rik*), −163/−163 (*F06Rik*)	8.3 ± 2.4	3	8	66	ND	9 wks
*Prss58*	ES	−2501/−2501	11.0 ± 1.2	3	21	221	21	8 wks
*Slfnl1*	EP	−3575/−3575	10.8 ± 1.0	3	28	252	25	8 wks
*Sppl2c*	EP	−4295/−4295	9.6 ± 1.5	3	23	221	23	11 wks
*Stpg3*	EP	−1732/−1732	9.8 ± 0.5	3	24	234	24	8 wks
*Tex33*	EP	−7986/−7986	10.6 ± 1.8	3	22	246	22	11 wks
*Tex36*	EP	−15135/−15135	7.9 ± 0.3	3	27	236	30	8 wks

ND indicates not determined.

### Statistical analyses

Statistical analysis was performed using a two-tailed unpaired t-test by Microsoft Office Excel (Microsoft Corporation, Redmond, WA, USA). *P* values less than 0.05 were considered significant. Data represent the mean ± standard deviation (SD).

## Results

### Candidate target genes are testis-enriched and conserved between mouse and human

**Figure 1:**
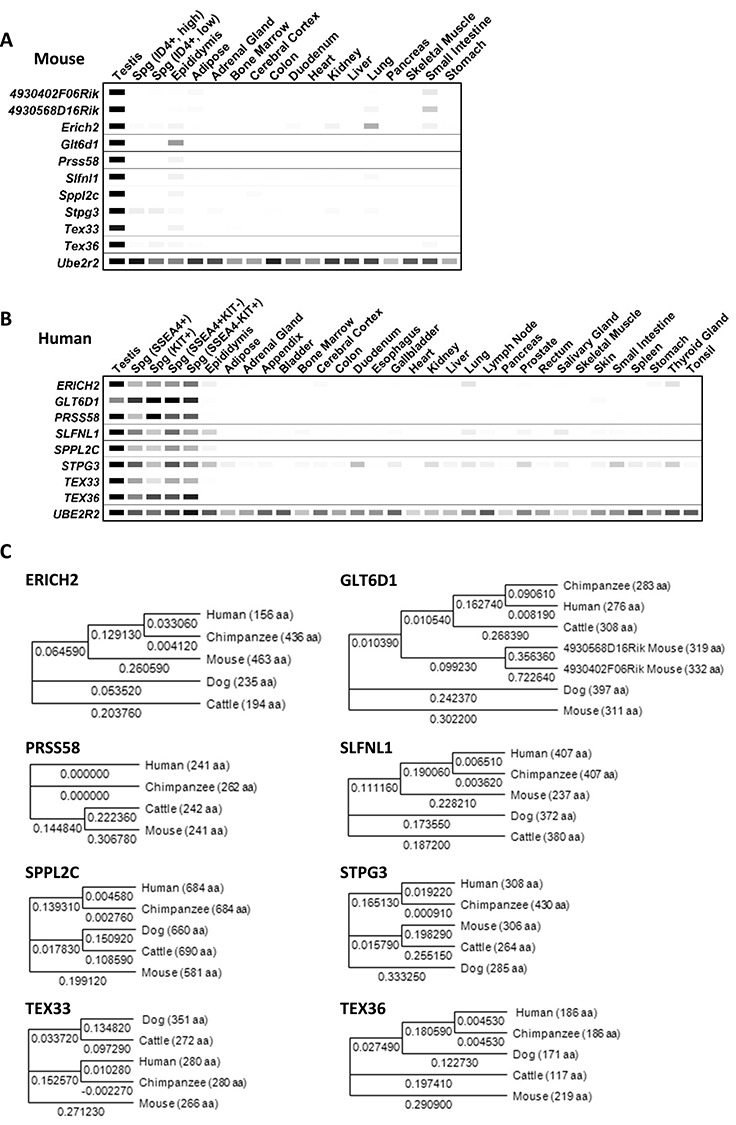
Candidate target genes are testis-enriched both in mouse and human and evolutionally conserved in mammals. (A and B) Digital PCR depicting the transcripts per million (TPM) value per tissue per gene from 240 published mouse and human RNA-seq datasets. (A) Gene expression patterns in mouse tissues. (B) Gene expression patterns in human tissues. (C) Phylogenetic trees of target genes in mammals. The number under the horizontal branches indicate relative branch lengths, and the number in parentheses indicates the length of the amino acid sequence.

We performed and compiled digital PCR data to confirm the expression of candidate target genes and revealed that these 10 genes in mouse (Figure 1A) and 8 orthologous genes in human tissues show testis-enriched expression (Figure 1B). Both *4930568D16Rik* and *4930402F06Rik* are shown only in the mouse because they are paralogs of *Glt6d1* (glycosyltransferase 6 domain containing 1) and not conserved in humans. To understand the evolutionary conservation of the candidate genes among mammals, we generated phylogenetic trees and found that all genes are highly conserved in mammals. When we generated a phylogenetic tree of GLT6D1, we added the mouse paralog proteins, 4930568D16Rik and 4930402F06Rik and found these mouse paralog proteins show more similarity to human GLT6D1 than to mouse GLT6D1 (Figure 1C); it is possible that 4930568D16Rik and 4930402F06Rik is the ortholog that then subsequently triplicated. To check the similarity of amino acid sequences, we compared the amino acid sequence of eight proteins between mouse and human and found that they are highly conserved between the two species (Supplementary Figure S1A–H). These results suggested that 8 of these 10 mouse proteins could be candidate targets for non-hormonal male contraceptive drugs in human.

### Generation of KO mice

To examine the essentiality of candidate target genes in male reproduction, we generated KO mouse lines using the CRISPR/Cas9 system. To generate both *Glt6d1* and *4930568D16Rik* single KO lines, we performed plasmid injections into the pronuclei of zygotes and obtained KO mouse lines that possess indel mutations individually. The embryo transfer efficiency (total pups divided by embryos transplanted) and CRISPR/Cas9 efficiency (the number of mutant pups divided by the number of genotyped pups) ranged from 13.0 to 27.0% and 28.6 to 50.0%, respectively. The efficiency of gene editing and mutation details using the zygote injection method is summarized in Supplementary Table S2.

For generating *Erich2*, *Slfnl1*, *Sppl2c*, *Stpg3*, *Tex33*, and *Tex36* single KO lines, two-pronuclear stage embryos were electroporated with guide RNA/Cas9 RNP. Gene KO mouse lines that possess a large deletion were obtained individually. The embryo transfer efficiency ranged from 12.8 to 50.9%, and CRISPR/Cas9 efficiency ranged from 30.0 to 79.3%. The efficiency of gene editing and mutation details using the zygote electroporation method is summarized in Supplementary Table S3.

The *Prss58* KO line was generated by the ES cell method. After transfection and selection of ES cells, 11 out of 32 clones possessed the *Prss58* KO allele, meaning 34.4% efficiency among the clones selected. We then obtained chimeric male mice by aggregating ES clones with 8-cell stage ICR embryos, and subsequent matings resulted in KO mutant mice. The efficiency of gene editing and mutation details using the ES cell method is summarized in Supplementary Table S4.

Because *Glt6d1* has two paralogs, *4930402F06Rik* and *4930568D16Rik* in mice, we expected that paralog proteins might compensate for each other in single KO mice. Therefore, we generated *Glt6d1*/*4930568D16Rik* DKO and *Glt6d1*/*4930568D16Rik*/*4930402F06Rik* TKO mouse lines. The DKO mouse line was generated by mating *Glt6d1* and *4930568D16Rik* single KO mice, and the TKO mice were generated by the zygote electroporation method using guide RNAs targeting *4930402F06Rik* and fertilized eggs obtained from IVF using DKO gametes. The mutation details are summarized in Supplementary Table S3.

### Phenotypic analysis of *Stpg3*, *Tex33*, and *Tex36* KO mouse lines

**Figure 2:**
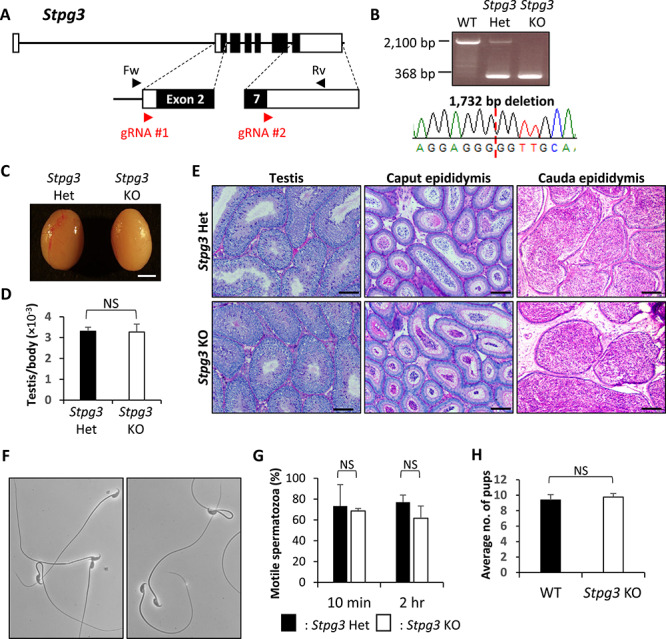
Phenotypic analysis of *Stpg3* KO male mice. (A) Genomic structure and KO strategy of targeting mouse *Stpg3*. Black boxes indicate exons and white boxes indicate non-coding sequences (Fw, forward primer for genotyping; Rv, reverse primer for genotyping). (B) Genotyping validation of *Stpg3* KO mice by PCR and Sanger sequencing. (C) Gross morphology of control and *Stpg3* KO testes. Scale bar is 2 mm. (D) Average weight of control and KO testis. Testis weight was divided by body weight. Student t-test, error bars represent S.D. (*n* = 6). NS indicates not significant. (E) Histological analysis with PAS staining of testis, caput, and cauda epididymis. Scale bars are 100 μm. (F) Morphology of sperm from control and KO mice collected from the cauda epididymis. Scale bars are 20 μm. (G) Motility of sperm from control and *Stpg3* KO mice. Motility was checked after 10 min and 2 h of incubation in TYH media. Student t-test, error bars represent S.D. (*n* = 3). NS indicates not significant. (H) Average litter size of WT and KO male mice. Student t-test, error bars represent S.D. (*n* = 3). NS indicates not significant.

**Figure 3:**
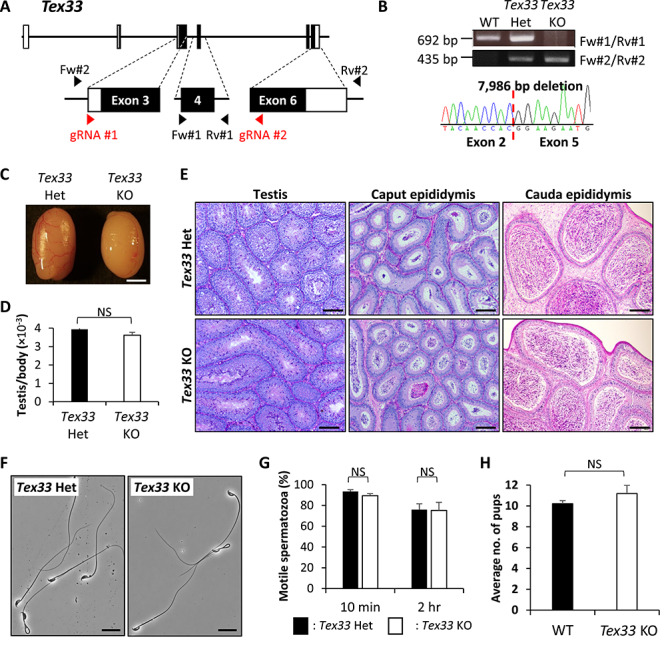
Phenotypic analysis of *Tex33* KO male mice. (A) Genomic structure and KO strategy of targeting mouse *Tex33*. Black boxes indicate exons and white boxes indicate non-coding sequences. (B) Genotyping validation of *Tex33* KO mice with PCR and Sanger sequencing. Two primer sets were used for genotyping PCR. (C) Gross morphology of control and *Tex33* KO testes. Scale bar is 2 mm. (D) Average weight of control and KO testis. Testis weight was divided by body weight. Student t-test, error bars represent S.D. (*n* = 3). NS indicates not significant. (E) Histological analysis with PAS staining of testis, caput, and cauda epididymis. Scale bars are 100 μm. (F) Sperm morphology of control and KO mice collected from cauda epididymis. Scale bars are 20 μm. (G) Sperm motility from control and *Tex33* KO mice. Motility was checked after 10 min and 2 h of incubation in TYH media. Student t-test, error bars represent S.D. (*n* = 3). NS indicates not significant. (H) Average litter size of WT and KO male mice. Student t-test, error bars represent S.D. (*n* = 3). NS indicates not significant.

**Figure 4:**
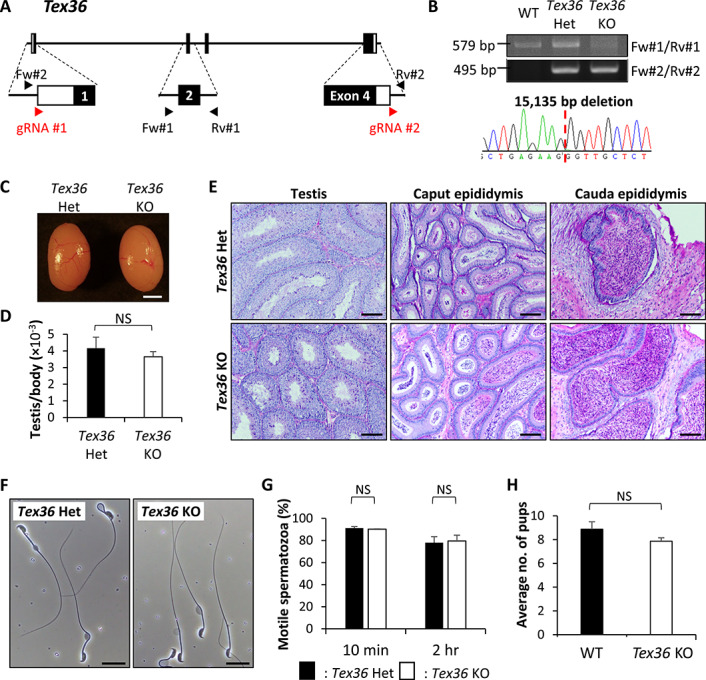
Phenotypic analysis of *Tex36* KO male mice. (A) Genomic structure and KO strategy of targeting mouse *Tex36*. Black boxes indicate exons and white boxes indicate non-coding sequences. (B) Genotyping validation of *Tex36* KO mice with PCR and Sanger sequencing. Two primer sets were used for genotyping PCR. (C) Gross morphology of control and *Tex36* KO testes. Scale bar is 2 mm. (D) Average weight of control and KO testis. Testis weight was divided by body weight. Student t-test, error bars represent S.D. (*n* = 3). NS indicates not significant. (E) Histological analysis with PAS staining of testis, caput, cauda epididymis. Scale bars are 100 μm. (F) Sperm morphology of control and KO mice collected from cauda epididymis. Scale bars are 20 μm. (G) Sperm motility from control and *Tex36* KO mice. Motility was checked after 10 min and 2 h of incubation in TYH media. Student t-test, error bars represent S.D. (*n* = 3). NS indicates not significant. (H) Average litter size of WT and KO male mice. Student t-test, error bars represent S.D. (*n* = 3). NS indicates not significant.

Phenotypic analyses of KO male mice were performed to examine both testicular and sperm development in the absence of testis-enriched genes of interest. We observed no abnormal development both in testis and spermatozoa in all generated KO lines used in the present study. Here, we show the results of *Stpg3*, *Tex33*, and *Tex36* KO lines as examples for in vivo functional analyses (Figures 2–4). We also show testicular histology and sperm morphology of *Sppl2c*, *Slfnl1*, and *Glt6d1/4930568D16Rik/4930402F06Rik* KO lines (Supplementary Figures S2 and S3).

For obtaining the *Stpg3* KO mouse line, we designed guide RNAs near both start and stop codons (Figure 2A). After generating KO mice, we performed genomic PCR using primers presented in Figure 2A and Sanger sequencing, revealing that the KO line has a 1,732 bp deletion (Figure 2B). Homozygous KO mice were viable and showed no overt abnormalities. Then, we performed the morphological and histological analysis of testes of *Stpg3* KO mice. There were no significant differences in gross appearance (Figure 2C), testicular weight (Figure 2D), nor testicular histology (Figure 2E) between control and KO mice. We also performed histological analysis of both caput and cauda epididymides in *Stpg3* KO male mice and found no significant differences (Figure 2E). To examine the effects on sperm development in the absence of *Stpg3*, we observed sperm morphology and found no overt differences (Figure 2F). Then, we checked the ratio of motile sperm in *Stpg3* KO mice using computer-assisted sperm analysis (CASA). The CASA revealed that the ratio of motile sperm in *Stpg3* KO mice was comparable with that of control (Figure 2G). Besides, *Stpg3* KO male mice can sire pups with comparable litter sizes as WT (Figure 2H).

To generate *Tex33* KO mice, we designed guide RNAs to exon 3 and exon 6 (Figure 3A). *Tex33* KO mice were generated by the zygote electroporation method, and their genotype were confirmed by genomic PCR using primers shown in Figure 3A. Sanger sequencing revealed that the KO line has a 7,986 bp deletion (Figure 3B). Homozygous KO mice were viable, and there were no observable abnormalities. The morphological and histological analyses of *Tex33* KO testes were then performed. There were no significant differences in appearance (Figure 3C), testis weight (Figure 3D), and testis histology using PAS staining (Figure 3E). Caput and cauda epididymis of *Tex33* KO male mice were also used for histological analysis, and there were no significant differences (Figure 3E). We observed the sperm morphology obtained from *Tex33* KO mice, but found no difference (Figure 3F). Using CASA, we checked the motile sperm ratio and found the ratio of KO male mice was comparable to that of control (Figure 3G). Also, litter size from KO males was comparable to control (Figure 3H).

The *Tex36* KO line was generated by using two guide RNAs designed upstream of the start codon in exon 1 and downstream of the stop codon in exon 4 (Figure 4A). PCR with two primer sets shown in Figure 4A and Sanger sequencing showed that the KO line has a 15,135 bp deletion (Figure 4B). We confirmed that homozygous KO mice were viable and their appearance was comparable to controls. We performed morphological and histological analysis of testes of *Tex36* KO male mice. There were no significant differences in appearance (Figure 4C), testis weight per body weight (Figure 4D) and testis histology (Figure 4E). We also performed histological analysis of caput and cauda epididymis of *Tex36* male mice, and there were no significant differences (Figure 4E). We then observed the spermatozoa to find any effect of the absence of *Tex36* but found no difference (Figure 4F). We checked motile sperm ratios using CASA and found that the ratio of KO was not different to that of control (Figure 4G). The average number of pups sired by KO male mice was also comparable to that of control (Figure 4H).

### Fertility tests of the remaining KO male mice

To check the effects on male fecundity in the absence of the other candidate target genes for non-hormonal male contraception (*Erich2*, *Prss58*, *Slfnl1*, *Sppl2c*, *Glt6d1*, *4930568D16Rik*, *Glt6d1*/*4930568D16Rik* DKO and *Glt6d1*/*4930568D16Rik*/*4930402F06Rik* TKO), we performed mating tests using all KO lines. The data show all KO male mice could sire pups with comparable litter size as WT controls (Table 1). Thus, the testis-enriched genes examined in this study are not required for male fecundity. All KO mouse lines except for the DKO mouse line were deposited as frozen spermatozoa to both the RIKEN BioResource Research Center (Riken BRC; Tsukuba, Japan) and the Center for Animal Resources and Development at Kumamoto University (CARD; Kumamoto, Japan). All lines are available to all researchers through these centers. The identification information for each knockout mouse line is summarized in Supplementary Table S6.

## Discussion

Although novel non-hormonal male contraceptive drugs are anticipated, very few have been developed and even of those few not all are satisfactory to all people [4]. To solve this problem, we searched for new candidate target proteins for non-hormonal contraceptive drugs. Using in silico analysis, we found that *Erich2*, *Glt6d1*, *Prss58*, *Slfnl1*, *Sppl2c*, *Stpg3*, *Tex33*, and *Tex36* show testis-enriched expression in both mouse and human (Figure 1A and B), and these coding protein genes are highly conserved in both (Supplementary Figure S1). Based on these findings, we hypothesized that these proteins could be targets of non-hormonal contraceptives. Thus, we generated gene KO mouse lines using the CRISPR/Cas9 system to analyze their roles in male reproduction. However, all KO male mice tested in this study show normal fecundity, indicating that these 10 genes are individually dispensable for male fertility (Table 1).

Among the candidate genes used in this study, signal peptide peptidase-like 2c (*Sppl2c*) KO mice have been already reported previously [28, 29]. In these papers, SPPL2C was suggested to be involved in acrosome formation during spermatogenesis [28] and to regulate Ca^2+^ homeostasis in spermatids [29]. Niemeyer et al. performed mating tests using *Sppl2c* KO male mice with WT female mice, and the litter size was comparable with WT males. However, *Sppl2c* KO intercrosses exhibited reduced litter sizes around four pups on average [29], indicating that the combination of male and female defects induces subfertility. Our results reconfirmed that *Sppl2c* KO male mice show normal fecundity, suggesting that SPPL2C cannot be a target for a male contraceptive, although this protein may play a limited function in spermatogenesis.

GLT6D1 is in the GT6 glycosyltransferases gene family, which includes the ABO blood group [30]. From this previous report, it was suggested that GT6 glycosyltransferases exhibit complex evolutionary patterns with multiple examples of both gene gain and loss in different mammalian species. *Glt6d1* shows testis-enriched expression in both mouse and human (Figure 1A and B), and the mouse has two paralogs, *4930568D16Rik* and *4930402F06Rik*, that are not conserved in human. At first, we generated both *Glt6d1* and *4930568D16Rik* single KO mouse lines individually to check their male fecundity and found that both KO male mice are fertile. We hypothesized that GLT6D1 and its paralogs compensate each other’s function in male reproduction. To remove this possibility, we generated *Glt6d1*/*4930568D16Rik* DKO and *Glt6d1*/*4930568D16Rik*/*4930402F06Rik* TKO mouse lines. However, the DKO and TKO male mice show normal fertility (Table 1), indicating that GLT6D1 in human is also dispensable for male fecundity.

Although our study revealed that 10 genes are not essential for male fertility under our mating conditions, these genes may still function in male reproduction such as *Sppl2c* [28, 29]. While further studies are needed for revealing protein functions, in vivo functional screens using CRISPR/Cas9 are a quick and accurate method to find genes that are essential for male fecundity. In the present study, we could not find any target proteins for non-hormonal male contraceptive drugs, but our findings will help to streamline other researchers’ efforts in focusing on essential genes for male fertility. We anticipate that target proteins and contraceptive drugs will be found using this method.

## Supplementary Material

SI_all_ioaa084

## Notes

**Grant support:** This work was supported by the Ministry of Education, Culture, Sports, Science and Technology (MEXT)/Japan Society for the Promotion of Science (JSPS) KAKENHI grants (JP20J10363 to S.P., JP17K17852 and JP20K16107 to K.S., JP16KK0180 to Y.F., JP17H01394 and JP19H05750JP to M.I.); Japan Agency for Medical Research and Development (AMED) grant JP19gm5010001 to M.I.; Takeda Science Foundation grants to Y.F. and M.I.; Senri Life Science Foundation grant to Y.F.; *Eunice Kennedy Shriver* National Institute of Child Health and Human Development (grants R01HD088412 and P01HD087157 to M.M.M. and M.I. and grant R01HD095341 to T.X.G.); the Bill & Melinda Gates Foundation (INV-001902 to M.M.M. and M.I.) and National Institute of General Medical Sciences (T32GM088129 and T32GM120011 to D.J.D.)
