# Supplementary material for: CRISPR/Cas9-mediated genome-edited mice reveal 10 testis-enriched genes are dispensable for male fecundity
Source: Biol Reprod. 2020 May 26;103(2):195–204. doi: 10.1093/biolre/ioaa084 (PMC7401030; doi:10.1093/biolre/ioaa084)
Supplement: SI_all_ioaa084 [file si_all_ioaa084.pdf]

Supplementary Figure S1 (Park *et al.*)

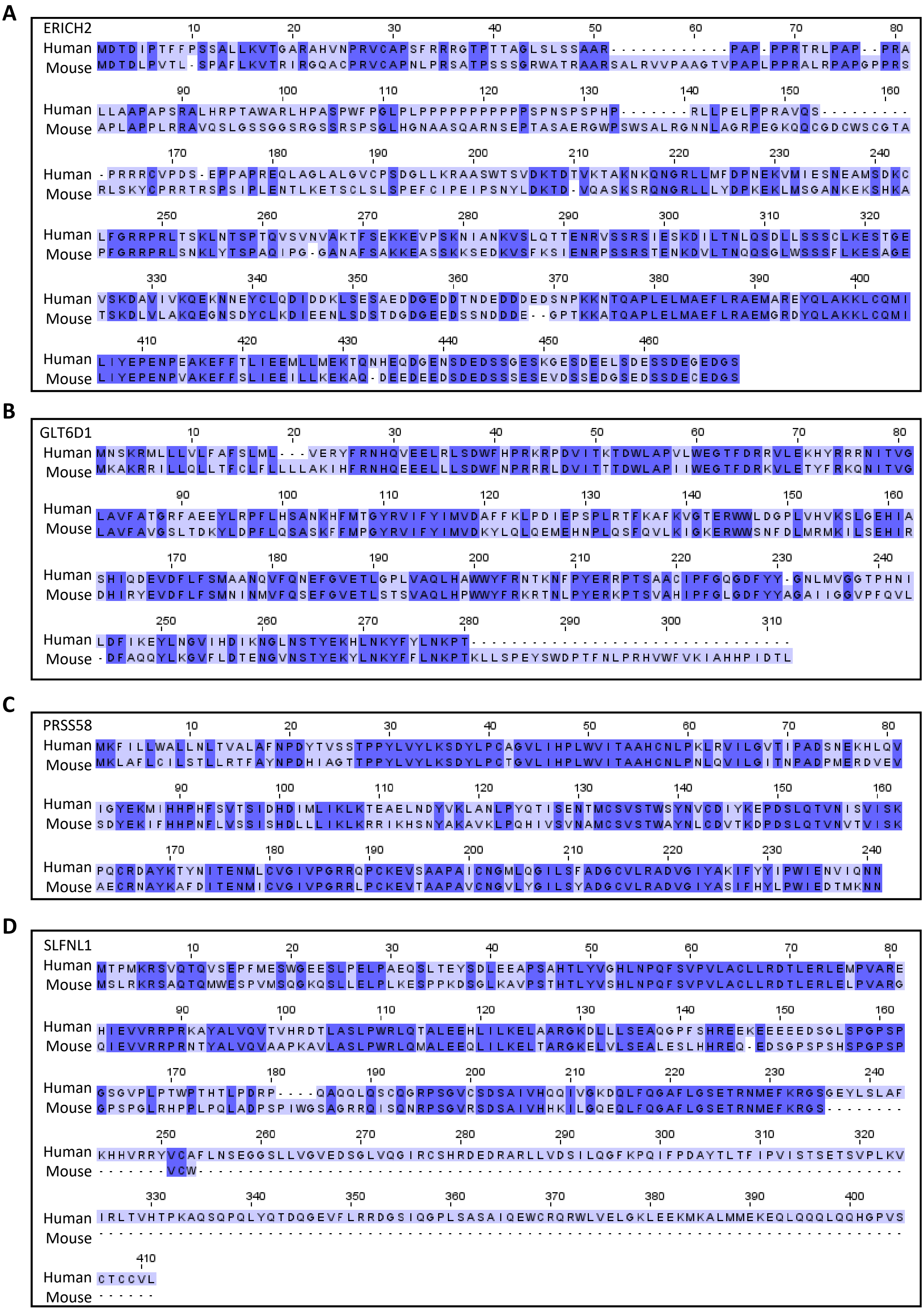

Supplementary Figure S1 (Park *et al.*)

E

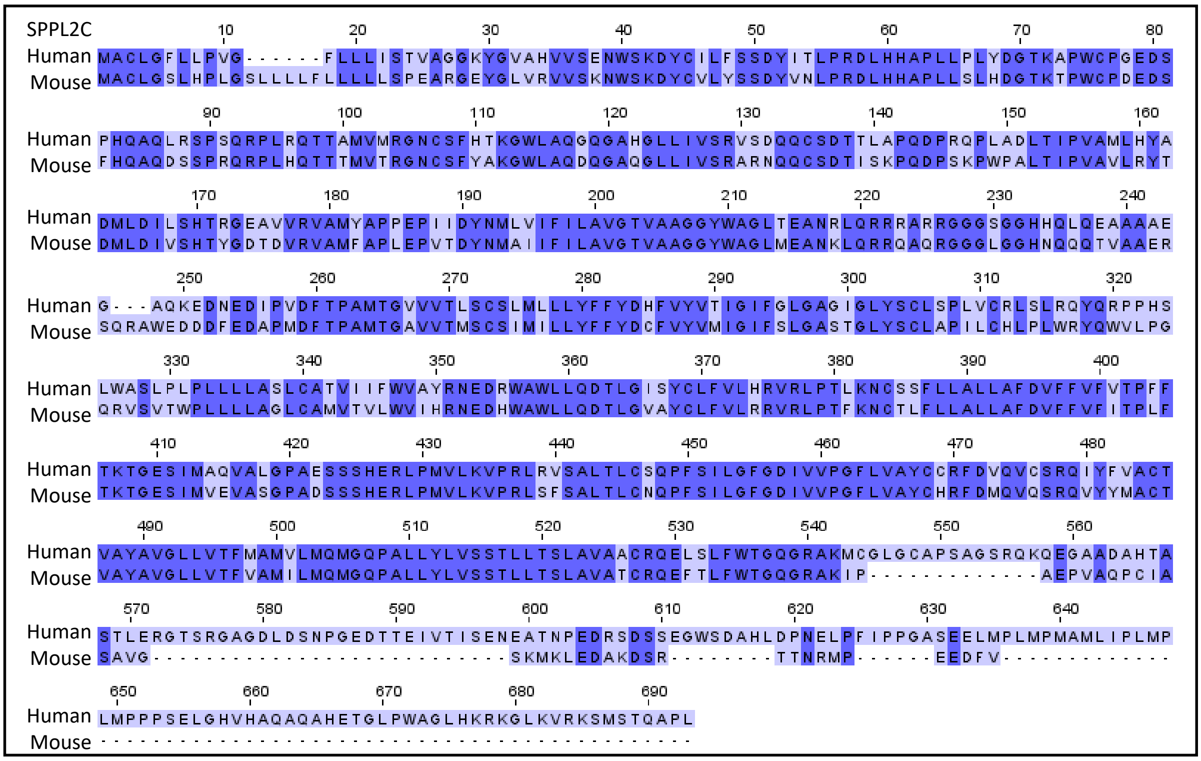

F

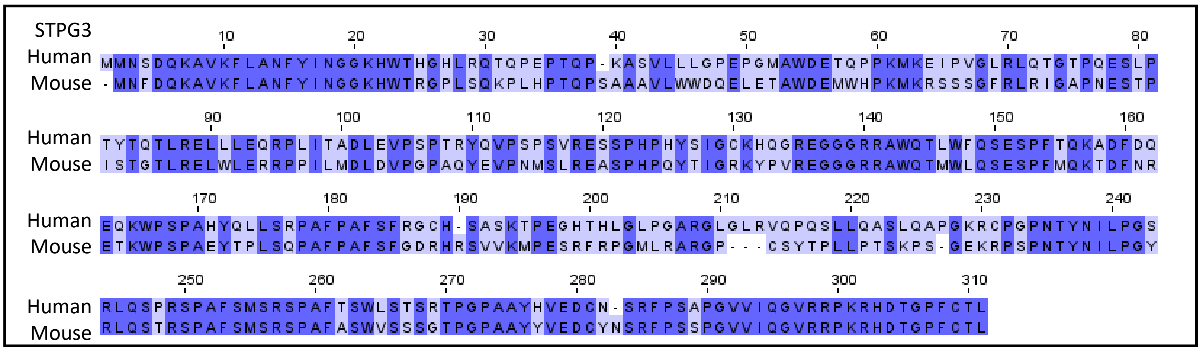

G

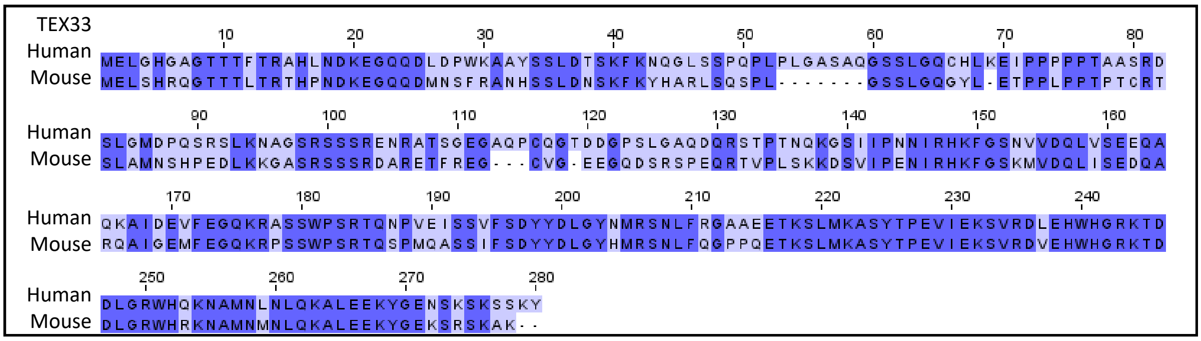

H

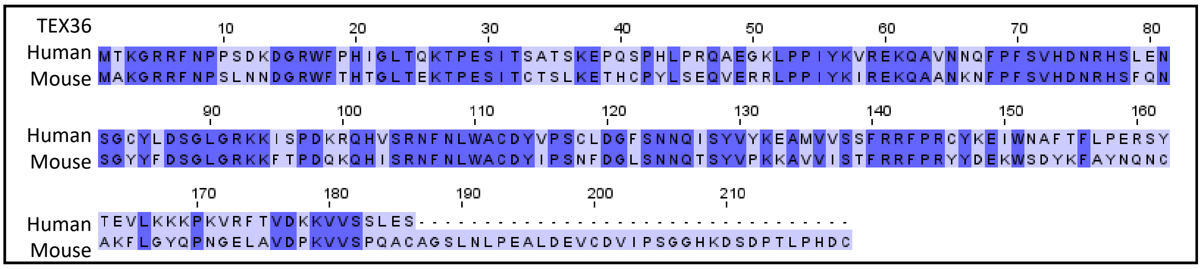

Supplementary Figure S2 (Park *et al.*)

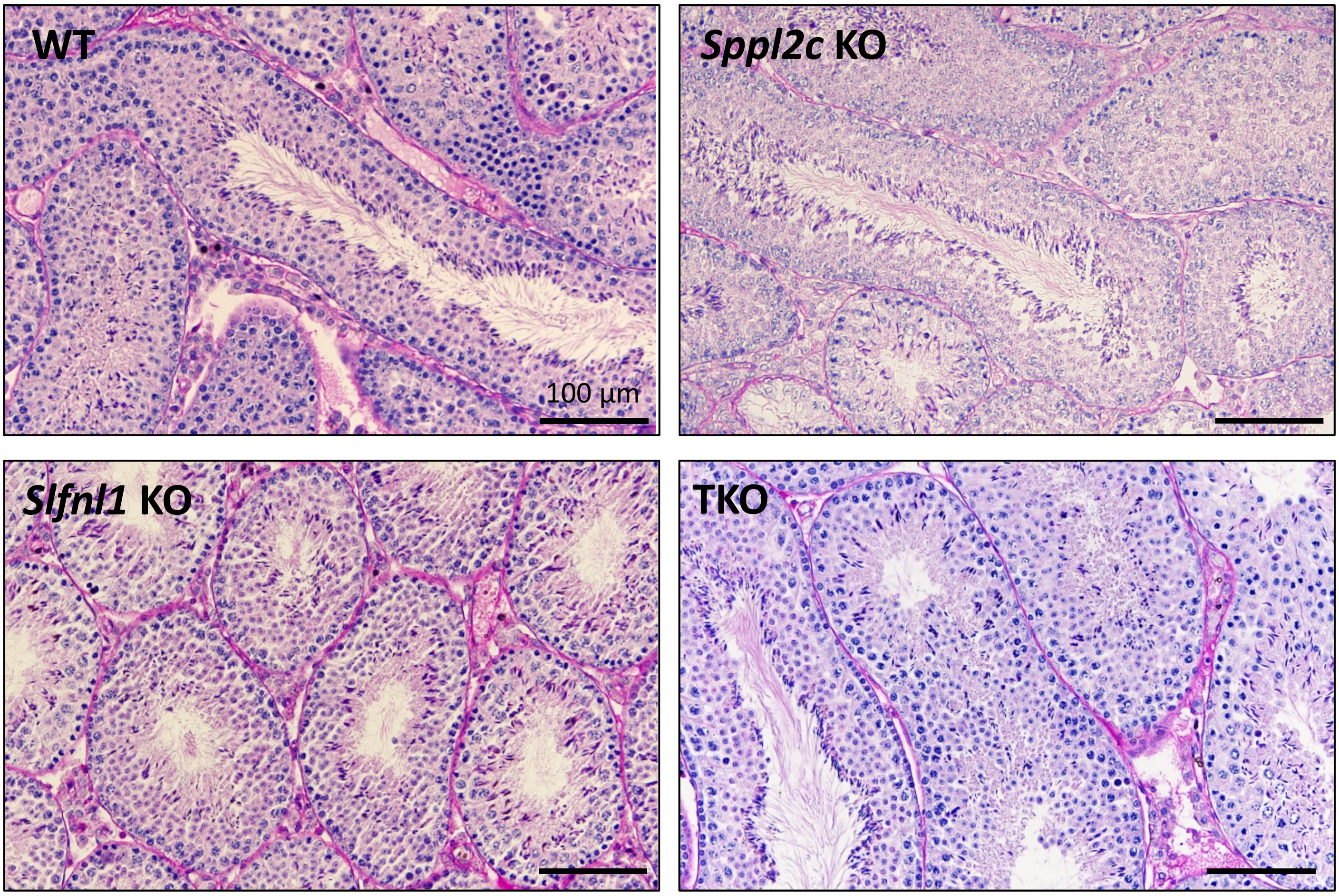

Supplementary Figure S3 (Park *et al.*)

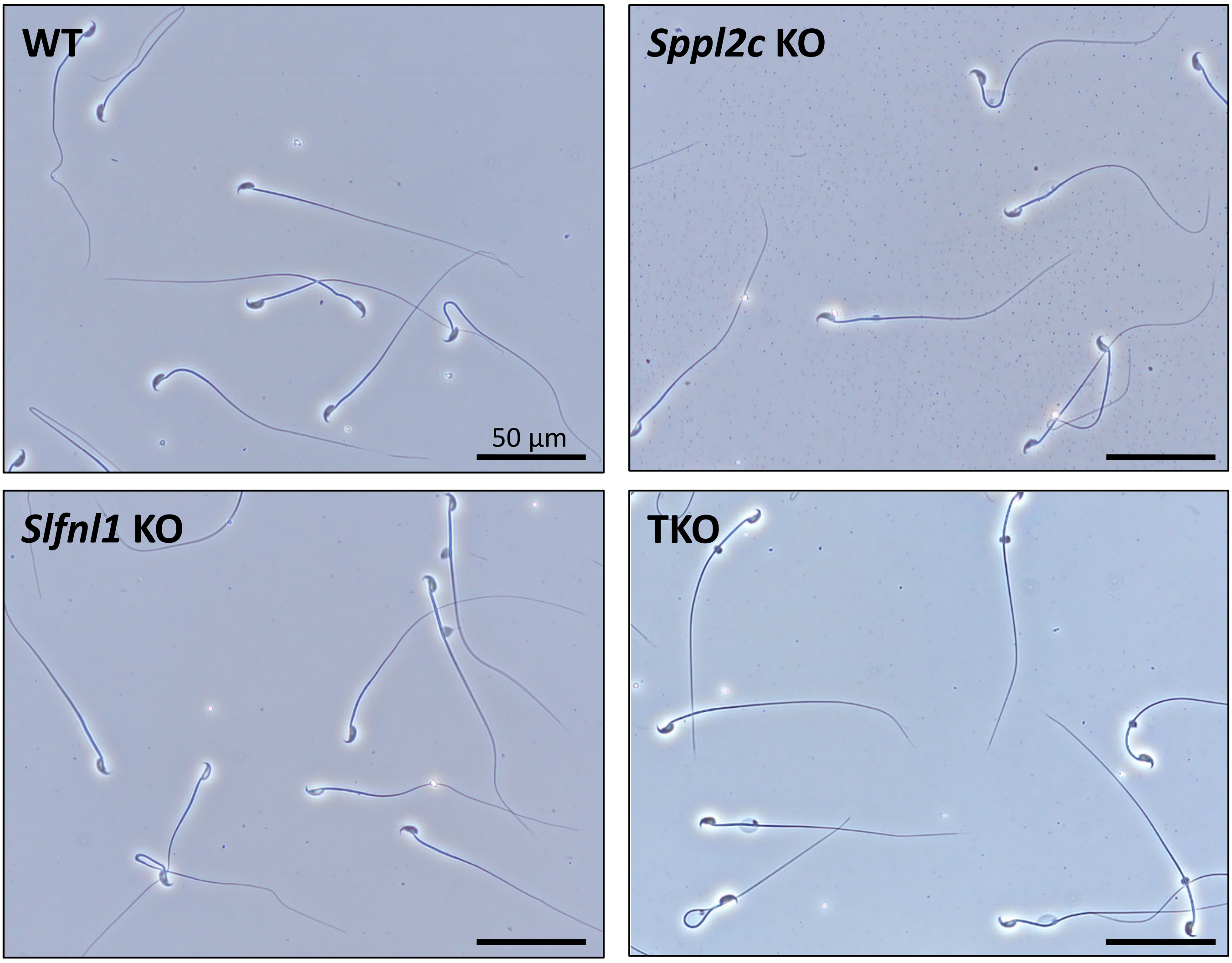

Supplementary Table S1 (Park *et al.*)

Protein symbol	Human sequence	Mouse sequence	Chimpanzee sequence	Cattle sequence	Dog sequence
4930402F06Rik	-	NP_001074178	-	-	-
4930568D16Rik	-	NP_083739	-	-	-
ERICH2	NP_001276876	NP_080020	XP_009442007	XP_024833663	XP_013966347
GLT6D1	NP_892019	NP_001034184	XP_016815992	NP_001069396	XP_013972426
PRSS58	NP_001001317	NP_778185	XP_016813776	NP_001035599	-
SLFNL1	NP_001161719	XP_006502944	XP_016789494	XP_024845982	XP_013974937
SPPL2C	NP_787078	NP_001076004	XP_523673	XP_002696104	XP_548046
STPG3	NP_001004353	NP_083237	XP_016817739	XP_024855460	XP_005625485
TEX33	NP_001157329	NP_001157084	XP_001145584	XP_024848033	XP_022280016
TEX36	NP_001121674	NP_082930	XP_003312864	NP_001070494	XP_022267639

Supplementary Table S2 (Park *et al.*)

Gene symbol	Guide RNA	Embryo transfer efficiency	CRISPR/Cas9 efficiency	CRISPR/Cas9 derived gene deletion (intron,EXON)	Sequence
Glt6d1	AGGAAGGAATGAAGGCTAAA	20/74 (27.0%)	10/20 (50.0%)	up: tcccacgcacaggaaggaATGAAGG<br>down: AGGATTTTATTACAATTACT	-8 bp
4930568D16Rik	CTCAACGCTTCTCCTACTCC	7/54 (13.0%)	2/7 (28.6%)	up: TGGCACTAACTCAACGCTTCTCCT<br>down: actatgtaaccctgaggtatcacagt	-10 bp

Supplementary Table S3 (Park *et al.*)

Gene symbol	Guide RNAs	Embryo transfer efficiency	CRISPR/Cas9 efficiency	CRISPR/Cas9 derived gene deletion (intron,EXON)	Sequence
Erich2	up : CAGCTGCTGGAACCACGCGA<br>down : AGCTCGGACGAGTGCGAGGA	16/69 (23.2%)	7/16 (43.8%)	up: CCCGCGCCGCCCGCTCCGCCCTTCG<br>down: GATGGGTCCTAGGGGGCATTGCTGC	-31,536 bp
4930402F06Rik (TKO)	up : GGGTAGATCTGAGTCGCCAT<br>down : ATTCAAAGCCAAGTACGGTC	29/57 (50.9%)	23/29 (79.3%)	up: TCCTATATGCCATGGGTAGATCTGAGTCGC<br>down: GTCTGGAGACTCTGGGAGACTCTG	-163 bp
Slfnl1	up : GTCTGAGAGCTAGGCCCAGG<br>down : AACTCTTGGCACATCCTCAA	9/37 (24.3%)	7/9 (77.8%)	up: CCTGGCCAGTCTGAGAGCTAGGCCC<br>down: tgctttcaggaaatttggctagtct	-3,575 bp
Sppl2c	up : GGACCAGGCCGTATTCTCCC<br>down : GTGTGAACAGCCTACGCTCC	20/60 (33.3%)	9/16 (56.3%)	up: TTCCTCCTCCTCCTCCTCAGCCCCG<br>down: AGCCTACGCTCCAGGACTCACTAGA	-4,295 bp
Stpg3	up : CAGGTAAGGAGGGGGTACTA<br>down : ACTATAGGCCCATCGGGTAT	11/86 (12.8%)	7/11 (63.6%)	up: tgacagtctcagcaggtaaggaggg<br>down: ggttgcaaagctgacctctcttgag	-1,732 bp
Tex33	up : CAGGTACAACCACGTTGACA<br>down : TAGCATTCTTCCGGTGCCAC	10/60 (16.7%)	3/10 (30.0%)	up: ttactccctgcccagGTACAACCAC<br>down: GGAAGAATGCTATGAACATGAACTT	-7,986 bp
Tex36	up : GAAGATCAGATACAGTGGGT<br>down : TCCAGCCCTATGATGTCTTG	16/36 (44.4%)	9/16 (56.3%)	up: ctctgcccaggcaacagctgagaag<br>down: ggttgctctttgttgtagggtaatt	-15,135 bp

Supplementary Table S4 (Park *et al.*)

Gene symbol	Guide RNAs	CRISPR/Cas9 efficiency	No. of injected ES cell clones	CRISPR/Cas9 derived gene deletion (intron,EXON)	Sequence
Prss58	up : AGCTATGTGATCTGGATTAT<br>down : AATAACTGAGCTCCCACAAC	11/32 (34.4%)	2	up: TTCTTTCCAGGTACCTTTGCCTATA<br>down: GGAGGAATTCTGAAACATGTGACAA	-2,501 bp

Supplementary Table S5 (Park *et al.*)

Gene symbol	Primer set for WT	Annealing condition	Elongation condition	Band size (bp)	Primer set for KO	Annealing condition	Elongation condition	Band size (bp)
4930402F06Rik	Fw:CCTTTTGTACTACTTGGGTCACTTCC<br>Rv:GGTGACAAGAGTTTAGTGGGTCTATGG	65°C<br>30 s	72°C<br>30 s	588	(as WT)	65°C<br>30 s	72°C<br>30 s	425
4930568D16Rik	Fw:CAAGACCTGCTTTTGATTGATGCCTGC<br>Rv:GCACAGGAAACAGGCTGTTTCTTTCC	65°C<br>30 s	72°C<br>30 s	687	(as WT)	65°C<br>30 s	72°C<br>30 s	677
Erich2	Fw:AATCTTCTCGGGGCGATCTC<br>Rv:CGTTGCCATGGAGACCGGAC	65°C<br>30 s	72°C<br>30 s	498	Fw:(as WT)<br>Rv:AACATGCTACATAGGGTTGT	65°C<br>30 s	72°C<br>30 s	604
Glt6d1	Fw:CAGCAAGTTCAAAGGCACTTCAAGCAG<br>Rv:GCACTGTCCTCTGCATGGCTCC	65°C<br>30 s	72°C<br>30 s	567	(as WT)	65°C<br>30 s	72°C<br>30 s	559
Prss58	Fw:TTGCTGTTATACTTTTGAAC<br>Rv:AGTTATATACAAGTTGCTGG	60°C<br>30 s	60°C<br>30 s	303	Fw:GCTTGCTCAACAACTGTTCC<br>Rv:CAACTGATACCCCTATTAGC	60°C<br>30 s	60°C<br>30 s	702
Slfnl1	Fw:AAGTCAGGAAACCCCAGAGA<br>Rv:AGTATGTGTGCTGGGAACCG	65°C<br>30 s	72°C<br>30 s	492	Fw:(as WT)<br>Rv:CATGGCTGACCAAGTGTCTT	65°C<br>30 s	72°C<br>30 s	569
Sppl2c	Fw:ATGAGGACCATTGGGCATGG<br>Rv:CCTCAGAACTCTCGCTGTGG	65°C<br>30 s	72°C<br>30 s	795	Fw:TAGCTGTACTGAGGCCTGGG<br>Rv:TTGTGGCCTAGGGCCCTAGT	65°C<br>30 s	72°C<br>30 s	1,012
Stpg3	Fw:GGAAAAGGGGTTGGGACAGG<br>Rv:CCACGCAGGAACAGAGAAAG	60°C<br>15 s	72°C<br>120 s	2,100	(as WT)	60°C<br>15 s	72°C<br>120 s	368
Tex33	Fw:CCCCCATCAGGCTCGACAGG<br>Rv:GTCACCATCGAGCTCCAGGG	65°C<br>30 s	72°C<br>30 s	692	Fw:GCCCTGTAGTCTGGGTCTGG<br>Rv:TCTCCCCGAGGACGTGGAGG	65°C<br>30 s	72°C<br>30 s	435
Tex36	Fw:TGGAAGGAATACTTCAAAGG<br>Rv:CCTCACACTACCACGGCTGG	55°C<br>30 s	72°C<br>30 s	579	Fw:ACAATGCTGAATCCATGAGG<br>Rv:GAGAGGGGATAGATTACAGG	60°C<br>30 s	72°C<br>30 s	495

Supplementary Table S6 (Park *et al.*)

Gene	Strain name	RBRC No.	CARD ID
4930568D16Rik	B6D2-*4930568D16Rik* ^{em1Osb}	9952	2507
TKO	B6D2-*Glt6d1* ^{em2Osb} *4930568D16Rik* ^{em1Osb} *4930402F06Rik* ^{em1Osb}	11059	2966
Erich2	B6D2-*Erich2* ^{em1Osb}	11060	2967
Glt6d1	B6D2-*Glt6d1* ^{em2Osb}	10117	2590
Prss58	STOCK *Prss58* ^{em1Osb}	10812	2792
Slfnl1	B6D2-*Slfnl1* ^{em1Osb}	11061	2968
Sppl2c	B6D2-*Sppl2c* ^{em1Osb}	11024	2931
Stpg3	B6D2-*Stpg3* ^{em1Osb}	11033	2940
Tex33	B6D2-*Tex33* ^{em1Osb}	11019	2926
Tex36	B6D2-*Tex36* ^{em1Osb} Tg(CAG/Su9-DsRed2,Acr3-EGFP)RBGS002Osb	11055	2962
